# Effect of flash-free polymeric adhesive on enamel surface after orthodontic bracket debonding under simulated cariogenic and gastric acid conditions

**DOI:** 10.3389/fdmed.2026.1789369

**Published:** 2026-04-23

**Authors:** Khaled M. Alzahrani, Abdulrahman Almalki, Refal Albaijan, Tarek Ahmed Soliman

**Affiliations:** 1 College of Dentistry, Prince Sattam Bin Abdulaziz University, Al-Kharj, Saudi Arabia; 2 Conservative Dental Sciences Department, College of Dentistry, Prince Sattam Bin Abdulaziz University, Al-Kharj, Saudi Arabia

**Keywords:** adhesive resin, demineralization, microhardness, oral environments, orthodontics

## Abstract

**Introduction:**

Enamel demineralization during orthodontic treatment may occur due to changes in the oral environments. This study aimed to assess the influence of flash-free polymeric adhesive systems on debonding behavior and subsurface enamel hardness under erosive and cariogenic conditions, compared with conventional orthodontic adhesives.

**Methods:**

Sixty premolars (*n* = 60) were randomly divided into two groups (*n* = 30 each): Group C, consisting of uncoated brackets, and Group F, comprising flash-free adhesive precoated brackets. Each group was further subdivided into three subgroups (*n* = 10) based on the storage medium used. In the artificial saliva subgroup (control group), specimens were stored in artificial saliva for 24 h. In the artificial cariogenic subgroup, specimens were alternately exposed to an artificial cariogenic solution and artificial saliva for 28 days. In the gastric acid subgroup, specimens were subjected to simulated gastric acid (pH = 1.2) by immersion for 2 min, six times daily, over a period of 9 days. All specimens underwent adhesive remnant index (ARI) testing and cross-sectional microhardness evaluation. Two-way ANOVA and Tukey's test were conducted to analyze cross-sectional microhardness. The chi-square test was used to analyze ARI scores.

**Results:**

Across all storage media, conventional brackets demonstrated higher frequencies of ARI score 1, whereas flash-free brackets consistently showed higher frequencies of ARI score 2. Across all storage media, the flash-free bracket system exhibited significantly higher overall microhardness (323.8 HV) compared with the conventional adhesive system (293.06 HV) (*p* < 0.05), indicating superior enamel protection under all tested conditions.

**Conclusions:**

Under cariogenic and erosive conditions, flash-free brackets demonstrated higher enamel cross-sectional microhardness and distinct adhesive remnant patterns. These findings suggest that flash-free polymeric adhesive systems may offer enhanced enamel protection during orthodontic treatment, particularly in patients exposed to cariogenic or gastric acid challenges.

## 1 Introduction

The preservation of intact enamel is essential for maintaining an aesthetically pleasing smile, and orthodontic interventions must preserve its original integrity. The application of fixed orthodontic appliances can lead to enamel demineralization both adjacent to and beneath bonded brackets (1–3). This demineralization reduces the bonding strength of brackets to tooth enamel (4), increases aesthetic concerns (5), and adversely affects the enamel surface properties such as smoothness and hardness (6). The evaluation of enamel alterations after orthodontic treatment has concentrated on demineralization occurring adjacent to and beneath orthodontic brackets (1, 2).

Enamel demineralization during orthodontic treatment may occur due to alterations in the oral environment caused by acid activity. These acids are produced either by bacteria, such as lactic, propionic, and butyric acids, or may originate from erosive sources, including acidic beverages (extrinsic sources) and conditions such as vomiting, regurgitation, or gastroesophageal reflux (intrinsic sources) (6–8). Previous studies have reported that enamel demineralization and the formation of white spot lesions in orthodontic patients, primarily due to bacterial acid activity, affect more than 45% of cases (7, 9). One contributing factor to this form of demineralization is the increased surface roughness caused by residual adhesive around brackets, which facilitates plaque accumulation (10, 11). Enamel erosion induced by acidic substances is a purely chemical process that occurs without bacterial involvement. Gastroesophageal reflux disease is considered a primary mechanism that exposes the oral cavity to gastric acid (12, 13). Previous studies have reported that gastroesophageal reflux disease affects approximately 10%–20% of the general population, with dental erosion observed in nearly 24% of affected individuals. Gastric acid, primarily composed of 0.1 M hydrochloric acid (HCl), poses a significant erosive challenge that may adversely affect the outcomes of orthodontic treatment (14).

Flash-free adhesive precoated brackets provide significant clinical advantages, such as shorter bonding time, minimal excess adhesive, and a smooth bracket–adhesive interface that may reduce plaque accumulation and, consequently, white spot lesion formation (15–18). These benefits are attributed to a unique polymer resin adhesive embedded within a non-woven polypropylene fiber material, which is precisely cut and applied to the bracket base. This adhesive is characterized by low filler content, low viscosity, and high wettability (15, 17). During the bonding process, the non-woven material facilitates extrusion of the adhesive, leaving a fillet margin around the bracket that is free of excess flash (18).

Previous investigations (16, 18–22) have indicated that flash-free, precoated bracket systems exhibit satisfactory clinical performance, particularly by reducing chairside time while preserving or improving bond strength. Despite the clinical advantages of flash-free polymeric adhesives, limited evidence exists regarding their ability to preserve enamel integrity following debonding, especially under combined cariogenic and erosive challenges. This study aimed to assess the influence of flash-free polymeric adhesive systems on debonding behavior and subsurface enamel hardness under erosive and cariogenic conditions, in comparison with conventional orthodontic adhesives. The evaluation focused on the adhesive remnant index and enamel cross-sectional microhardness. The null hypothesis proposed that there would be no significant differences between enamel surfaces after debonding with flash-free precoated brackets and those with traditionally coated brackets in terms of (1) the adhesive remnant index and (2) cross-sectional microhardness.

## 2 Methodology

This study evaluated two types of ceramic brackets based on their polymeric resin adhesive systems. One group comprised precoated ceramic brackets (Clarity™ Advanced, 3M Unitek, Monrovia, CA, USA, lot no. HU5ZX) featuring flash-free adhesive, while the other group included traditional ceramic brackets (Clarity™ Advanced, 3M Unitek, Monrovia, CA, USA, lot no. JV6QR) that were manually coated during bonding and served as the control group. It should be noted that the APC™ Flash-Free adhesive differs in formulation from Transbond™ XT, incorporating a low-viscosity resin embedded within a non-woven polypropylene fiber matrix. Therefore, this study compares two clinically integrated bonding systems rather than identical adhesive chemistries.

### 2.1 Specimen selection

The sample size was determined based on previously published *in vitro* orthodontic studies employing comparable experimental designs (23, 24). A total of 60 (*n* = 60) recently extracted premolars with intact buccal enamel were obtained from orthodontic patients undergoing tooth extraction at the College of Dentistry clinics, Prince Sattam Bin Abdulaziz University. Extracted teeth were obtained after written informed consent, anonymized, and used solely for laboratory research purposes. The premolars were preserved in a 0.1% thymol solution at pH 7.0 for 24 h to prevent bacterial contamination. The inclusion criteria included intact enamel with no fractures, cracks, or pre-existing demineralization. The premolars were cleansed with a pumice slurry and then preserved in distilled water at ambient temperature, with regular changes of the solution to prevent dehydration.

### 2.2 Study design and grouping

**Figure 1:**
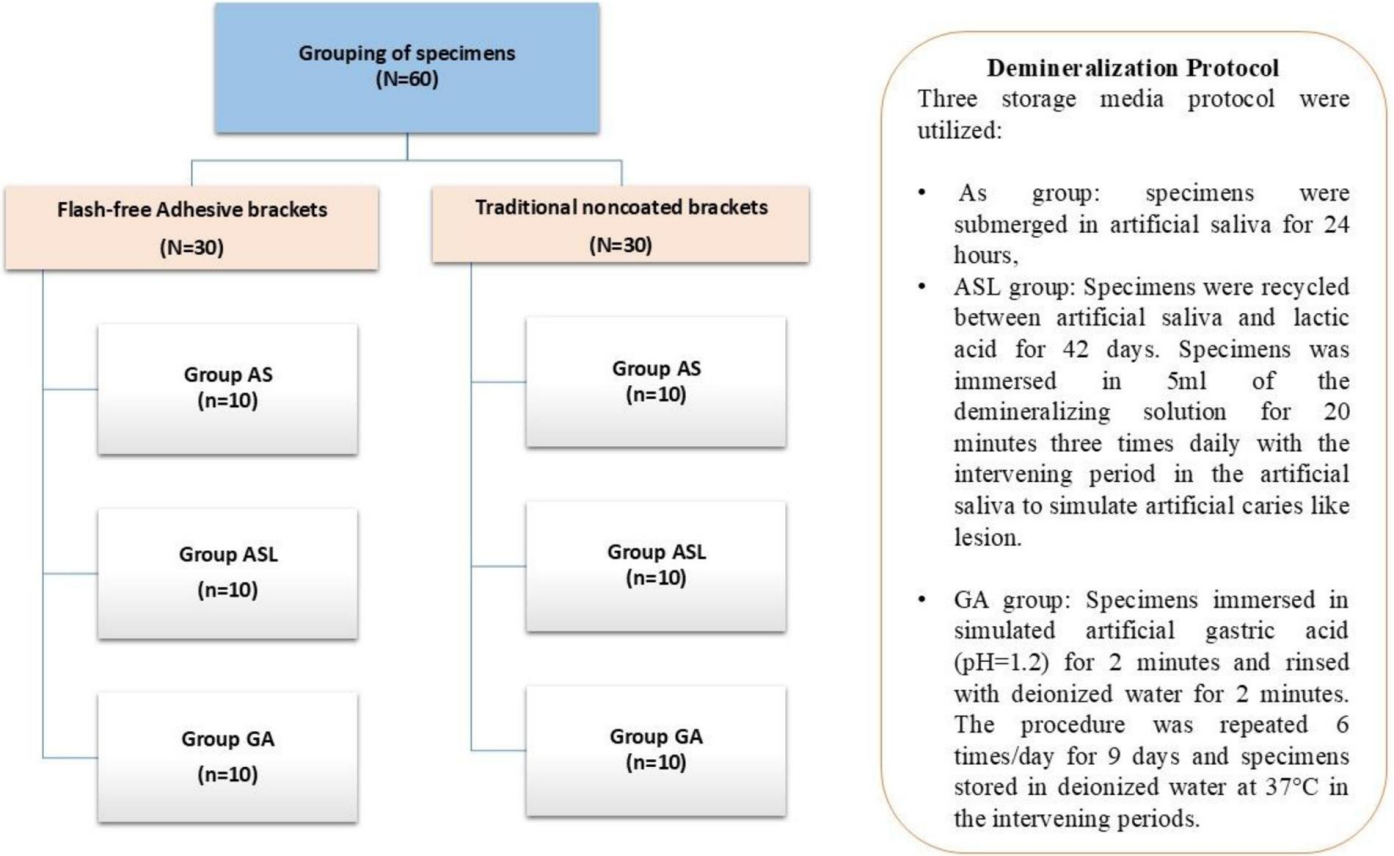
Study design and specimens: grouping.

Sixty premolars (*n* = 60) were randomly allocated into two groups (*n* = 30 each): Group C, comprising uncoated orthodontic brackets, and Group F, consisting of flash-free adhesive precoated brackets. Each group was further subdivided into three subgroups (*n* = 10) based on the type of storage medium employed: artificial saliva group, artificial cariogenic group (ASL), and simulated gastric acid group (GA) (Figure 1).

### 2.3 Enamel–bracket bonding

All groups had the buccal surfaces of the premolars conditioned using a self-etching primer (Transbond Plus, 3M Unitek, USA). Brackets were bonded by a single operator following the protocol specific to each adhesive system. For the adhesive precoated brackets, the blister tab lids were removed, and the brackets were gently positioned on the tooth surface before being firmly pressed in their final position. To standardize bracket seating and reduce variability in adhesive layer thickness, a 0.5-kg customized metallic tool was applied to the center of the bracket top surface. The applicator tip was flat-ended and positioned perpendicular to the buccal enamel surface at the center of the bracket base. A constant force was maintained for 10 s during initial positioning prior to light curing. This procedure was applied to all specimens to ensure a standardized pressure and uniform adhesive thickness at the enamel–bracket interface.

In the traditional bracket group, excess adhesive resin was removed using an explorer. An acid-resistant varnish was then applied to the enamel, leaving a 1-mm margin occlusal and apical to each bracket. For the traditional bracket group, Transbond™ XT composite (3M Unitek, Monrovia, USA) was utilized. All specimens were light-cured for 12 s from two directions, 6 s per direction, using an Ortholux Luminous Curing Light (3M Unitek; Monrovia, California, USA; light output: 1600 mW/cm^2^). The specimens were stored in distilled water at 37°C for 24 h to ensure complete polymerization (25).

### 2.4 Demineralizing protocols

Three storage media protocols were utilized. In subgroup AS (control group), specimens were stored in artificial saliva for 24 h. The artificial saliva consisted of 20 mmol/L NaHCO_3_, 3 mmol/L NaH_2_PO_4_, and 1 mmol/L CaCl_2_ at neutral pH.

In subgroup ASL, specimens were alternately exposed to an artificial cariogenic solution and artificial saliva for 28 days. Each specimen was immersed in 5 mL of lactic acid for 20 min, three times daily, with intervening period in artificial saliva to simulate an artificial caries-like lesion. The demineralizing solution consisted of 3.0 mmol/L CaCl_2_, 1.8 mmol/L KH_2_PO_4_, 0.1 mol/L lactic acid, and 1% carboxymethyl cellulose, with the pH adjusted to 4 using KOH (24, 26).

In subgroup GA, specimens were subjected to simulated gastric acid (pH = 1.2) by immersion for 2 min, six times daily, over a period of 9 days. During the intervening periods, specimens were stored in distilled water at 37°C (12, 13). All solutions were maintained at 37°C to replicate intraoral conditions and were replaced daily to ensure stable pH levels.

### 2.5 Bracket debonding and adhesive remnant index testing

Following exposure to the demineralization protocol, the brackets were deboned using bracket removal pliers. Bilateral pressure was carefully applied by gently compressing the mesial and distal wings of each bracket to minimize the risk of cracks or fractures on the buccal enamel surface. Following debonding, residual adhesive on the enamel surfaces was evaluated using an optical stereomicroscope (Olympus SZ61, Tokyo, Japan) at 30× magnification. The evaluation was performed using the following criteria (27): 0 = no adhesive present on enamel; 1 = less than 50% of adhesive remaining on enamel; 2 = more than 50% of adhesive remaining on enamel; 3 = all adhesive remaining on enamel.

### 2.6 Cross-sectional microhardness testing

**Figure 2:**
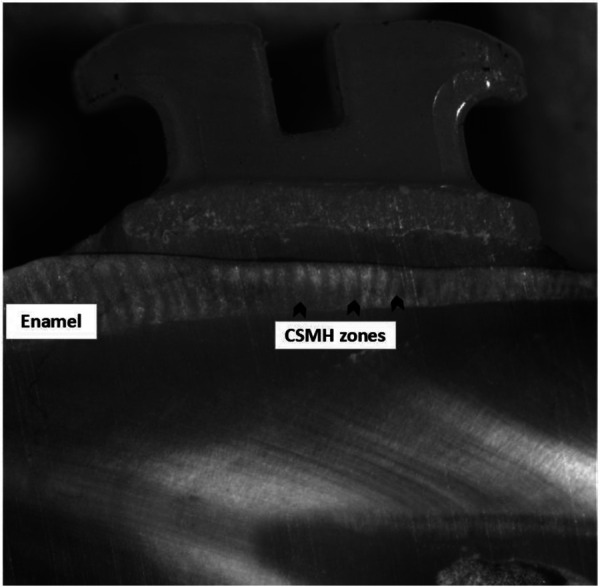
Cross-sectional micrograph showing the bracket–enamel interface and the CSMH indentation zones beneath the enamel surface for microhardness assessment.

Microhardness testing was utilized as an indirect measure of the degree of enamel demineralization. After the bracket bonding procedure, the specimens were sectioned sagittally through the center of the bracket into mesial and distal halves using a water-cooled, low-speed diamond saw (TechCut4, Allied, USA). The sectioned specimens were placed in acrylic resin blocks with the cut face exposed and subsequently polished using abrasive papers of progressively finer grit (320, 600, and 1,200 grit). Final refinement was performed using a 1-μm diamond paste utilized with a polishing cloth disc (Buehler). Microhardness was evaluated using a digital Vickers microhardness tester (HXD-1000 TM/LCD, Shanghai Optical Instrument Co. Ltd., Shanghai, China) with a load of 50 g and a dwell time of 15 s. Indentations were made on the buccal enamel surface beneath the bracket at a depth of 30 µm from the external enamel surface (16, 23). For each specimen, three measurements were recorded and averaged to determine the representative microhardness value (Figure 2).

### 2.7 Statistical analysis

The Shapiro–Wilk test (*p* ˃ 0.05) and Levene's test confirmed the normality and equal variance assumptions. A chi-square (*χ*^2^) test was employed to identify significant differences (*p* < 0.05) in adhesive remnant index (ARI) scores. Cross-sectional microhardness was analyzed using two-way ANOVA, followed by Tukey's *post-hoc* test for multiple comparisons. The significance level for all statistical tests was 5%.

## 3 Results

### 3.1 Adhesive remnant index

**Figure 3:**
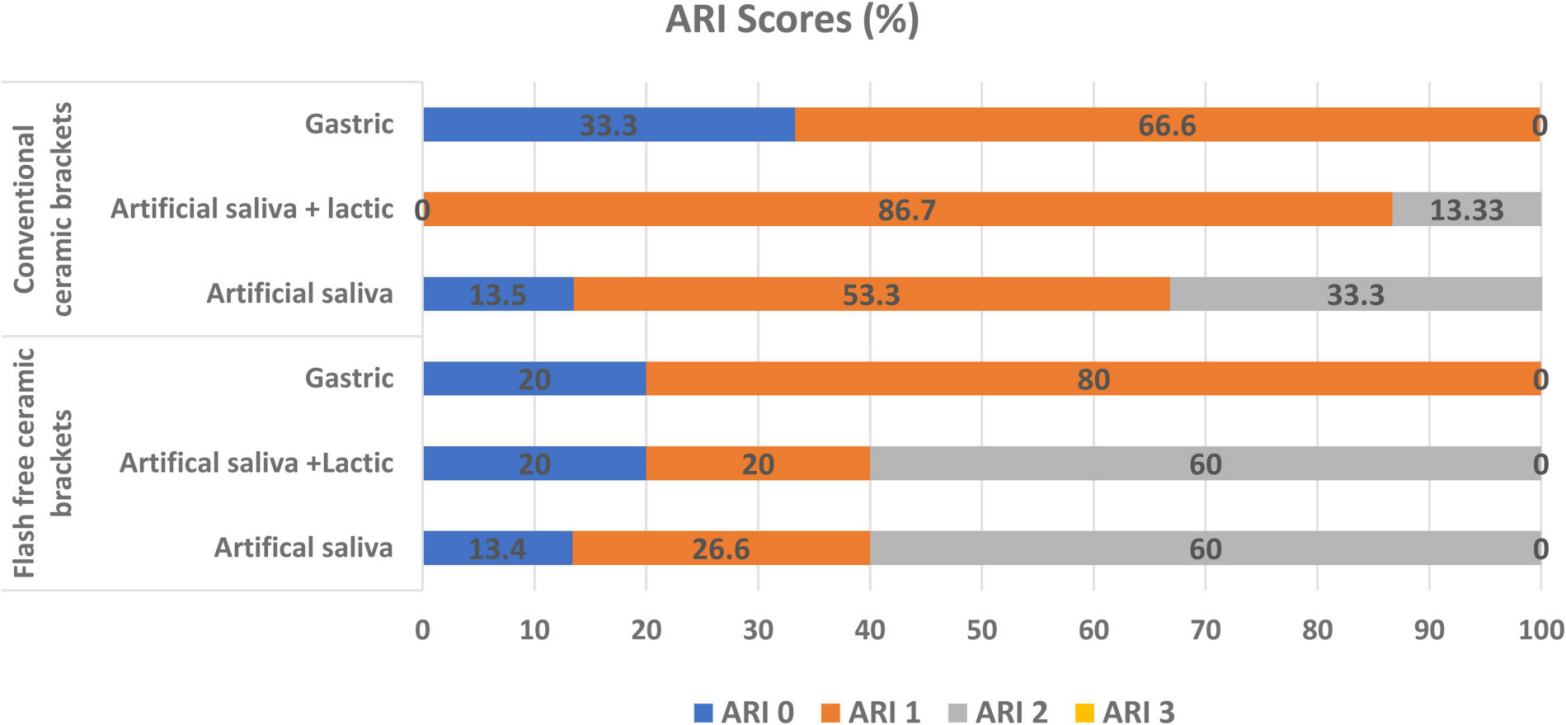
Percentage distribution of ARI scores for each subgroup.

**Figure 4:**
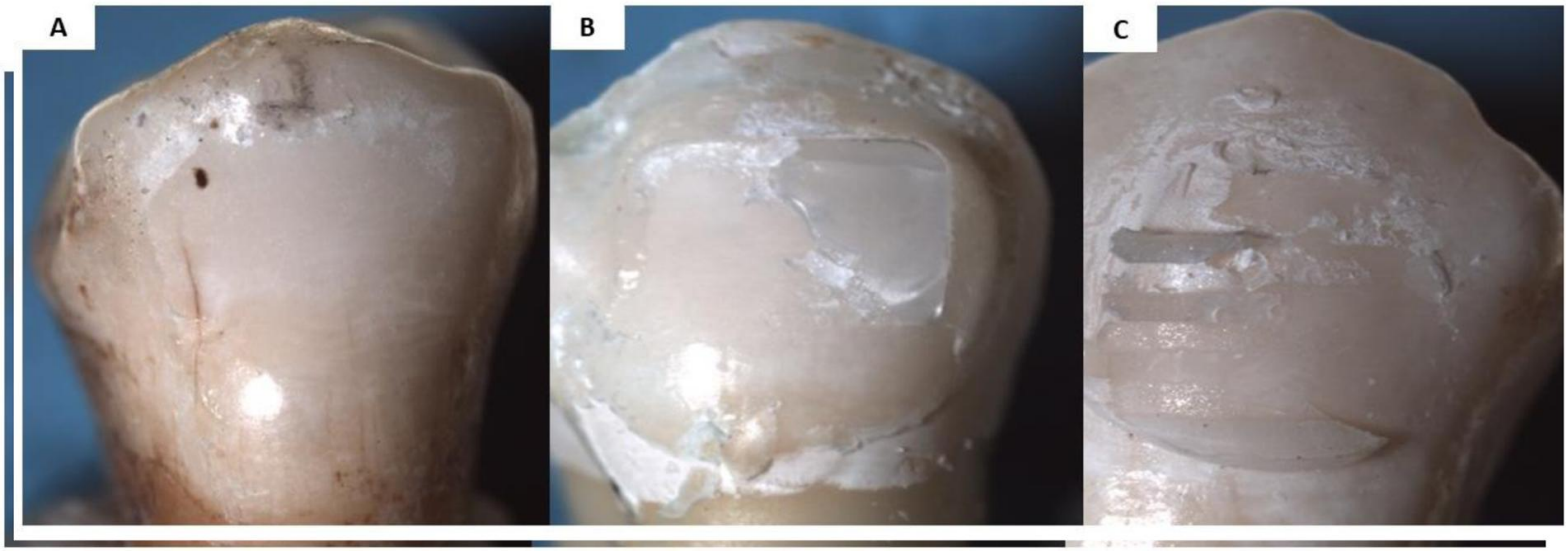
ARI scores for different groups. **(A)** ARI score 0; **(B)** ARI score 1; **(C)** ARI score 0.

Chi-square analysis revealed a statistically significant difference in ARI score distribution between the two bracket systems across all storage conditions (*p* < 0.05). Figure 3 illustrates the percentage distribution of ARI scores for each subgroup, while Figure 4 presents the ARI scores for different groups.

In the conventional bracket group, the majority of specimens in the AS group exhibited ARI score 1 (53.3%). In the ASL group, the majority of specimens demonstrated ARI 1 (86.7%), with only 13.3% showing ARI score 2 and no instances of ARI scores 0 or 3. Following exposure to gastric acid, most specimens demonstrated ARI score 1 (66.6%), while 33.3% showed ARI score 0. In the flash-free adhesive group, ARI score 2 was predominant (60%) in the AS group. In the ASL group, the majority of specimens demonstrated ARI score 2 (60%), with ARI scores 0 and 1 each observed in 20% of specimens. After gastric acid exposure, ARI score 1 was observed in 80% of specimens, with the remaining 20% showing ARI score 0. Across all storage media, conventional brackets demonstrated higher frequencies of ARI score 1, whereas flash-free brackets consistently showed higher frequencies of ARI score 2. These findings indicate statistically distinct failure profiles between the two adhesive systems, with the amount of adhesive remnant significantly influenced by both bracket type and storage medium (*p* < 0.05).

### 3.2 Cross-sectional microhardness

Cross-sectional microhardness values differed significantly among storage media and between the two bracket types (two-way ANOVA, *p* < 0.05). Table 1 summarizes the mean cross-sectional microhardness (CSMH) values and statistical comparisons. In the AS group, both types of orthodontic brackets showed the highest enamel hardness compared with other storage media. Flash-free brackets exhibited significantly greater CSMH values (386 HV) than conventional brackets (317.1 HV) (*p* < 0.05) in the AS group.

**Table 1:** Cross-sectional microhardness for the different groups.

Storage media groups	Orthodontic brackets	
	Conventional ceramic bracket	Flash-free ceramic bracket
AS	317.1 ± 32.45^Ab^	386 ± 35.28^Aa^
ASL	286.2 ± 25.89^Bb^	326.5 ± 29.45^Ba^
G	275.9 ± 27.45^BCc^	298.9 ± 24.34^Cc^
Overall CSMH	293.06 ± 29.78^Bb^	323.8 ± 27.39^Aa^

Mean values represented with different superscript lowercase letters (row) are significantly different according to Tukey's significant different test (*P* < 0.05). Mean values represented with different superscript uppercase letters (column) are significantly different according to Tukey's significant different test (*P* < 0.05).

Exposure to the artificial cariogenic protocol (ASL) resulted in a significant reduction in enamel hardness for both groups; however, enamel bonded with flash-free brackets maintained higher hardness (326.5 HV) than enamel bonded with conventional brackets (286.2 HV) (*p* < 0.05). Exposure to gastric acid (G) produced the greatest decrease in enamel hardness. Although both groups showed marked reductions, flash-free brackets again demonstrated significantly higher hardness (298.9 HV) than conventional brackets (275.9 HV) (*p* < 0.05). Across all storage media, the flash-free bracket system yielded significantly higher overall microhardness (323.8 HV) than the conventional adhesive system (293.06 HV) (*p* < 0.05), suggesting enhanced enamel preservation under controlled laboratory conditions.

## 4 Discussion

Orthodontic adhesives are considered one of the factors that can influence the techniques for attaching and removing orthodontic brackets. Advancements in adhesive systems have enhanced the bonding between enamel and resin; however, increased adhesion could complicate the debonding process. Furthermore, preventing demineralization beneath orthodontic brackets is essential for maintaining oral health and ensuring the effectiveness of orthodontic treatment. Therefore, this study aimed to evaluate the integrity of enamel surfaces following the debonding of ceramic brackets using two distinct polymer-based adhesive systems across two simulated clinical scenarios.

This study simulated two clinical scenarios. The first scenario replicated the cariogenic environment experienced by adhesive–enamel-bonded areas *in vivo*. Lactic acid (pH 4) was chosen as the demineralizing solution, as it is the principal acid produced by plaque microorganisms. A 28-day experimental period was chosen because demineralization occurs within approximately 1 month after bracket installation (27). The exposure duration (20 min, three times daily for 28 days) was designed to simulate repeated acid challenges interspersed with intermittent remineralization phases in artificial saliva, consistent with established *in vitro* pH cycling and artificial caries models (28). The second scenario simulated oral conditions in patients with GERD (gastroesophageal reflux disease) or bulimia nervosa, rather than average reflux patterns who vomit 6–10 times daily (29). The 2-min demineralization (acid attack) duration corresponded with the decrease in salivary pH during such acid attack (12, 27). Following every 2-min exposure, specimens were immersed in artificial saliva for 2 h to promote remineralization (27). This approach was selected to allow a standardized and accelerated assessment of material performance under reproducible laboratory conditions. Therefore, this model represents a controlled simulation of potential interfacial diffusion and adhesive degradation rather than direct clinical exposure.

Standardizing the seating pressure during bracket placement is essential, as the magnitude of the applied force directly influences the thickness of the adhesive layer at the enamel–bracket interface, which may affect stress distribution and the resulting ARI failure pattern. Previous studies have shown that varying bracket placement force can influence bonding outcomes by altering adhesive thickness (30–32). Therefore, a constant seating force was applied in the present study to reduce procedural variability and enable a more reliable comparison of debonding patterns between the bonding systems. An important factor in selecting orthodontic adhesives is the amount of residual adhesive after debonding, which can be determined using the ARI scoring system. The ARI score was used to determine the site of failure at the deboned interface (23). Both bracket type and storage medium had a significant impact on ARI scores, leading to the rejection of the null hypothesis. It is also important to consider that the flash-free system utilizes a distinct adhesive formulation compared with Transbond™ XT, which may contribute to the observed differences in failure patterns; however, this reflects the inherent design of each commercially available bonding system.

Following 28 days of exposure to the demineralizing solution, ARI scores were significantly reduced (indicating less adhesive residue on the enamel) compared with those in the artificial saliva group, indicating that the acidic environment markedly affected the adhesive–enamel bonding interface. Conventional brackets demonstrated ARI score 1 across all storage conditions, indicating that bond failure predominantly occurred at the bracket–adhesive interface. The findings align with previous studies (15, 16), which reported higher ARI scores for flash-free adhesive systems than conventional brackets.

Failure of flash-free adhesive brackets was primarily recorded with an ARI score of 2. While this failure pattern may increase chairside cleanup time, it is often associated with a reduced risk of enamel fracture during debonding. Compared with conventional brackets, the higher ARI scores may be attributed to the incorporation of a non-woven polypropylene fiber matrix within the bracket base, which allows controlled adhesive flow and uniform resin distribution during bracket placement. In addition, the low-viscosity, low-filler resin formulation may improve enamel wetting and penetration, resulting in a more stable enamel–adhesive interface and greater adhesive remnants remaining on the enamel after debonding. This design may improve micromechanical retention and promote cohesive failure within the adhesive layer within the adhesive failure at the enamel interface (25, 33, 34). After exposure to gastric acid, a noticeable shift toward lower ARI scores was observed in the flash-free group, with ARI score 1 becoming predominant. This change suggests that acidic conditions may compromise the cohesive integrity of the adhesive layer or reduce the effectiveness of resin–enamel bonding through hydrolytic degradation and plasticization of the adhesive matrix. Our findings are consistent with previous studies showing that gastric acid alters orthodontic bonding behavior and debonding patterns. Camcı et al. (34) reported that exposure to gastric acid negatively affected bond performance and resulted in measurable differences in ARI outcomes across bracket–adhesive systems. Similarly, Çınar et al. (35) used simulated gastric juice with thermocycling as a degradation model and confirmed that low-pH storage represents a clinically relevant challenge capable of influencing bonding outcomes and substrate–adhesive interaction.

Cross-sectional microhardness testing was used as an indirect measure to assess changes in enamel demineralization and remineralization. A penetration depth of 30 μm was selected for this study based on previous studies (23). The results of this study showed that both the storage medium and the type of orthodontic bracket system significantly influenced enamel cross-sectional microhardness, leading to rejection of the second null hypothesis. Enamel cross-sectional microhardness was the highest in the AS group compared with other demineralizing solution groups. Previous studies (24, 36) have demonstrated that demineralization leads to the loss of calcium and phosphate ions, subsequently influencing the hardness of the enamel.

Exposure to the artificial cariogenic protocol resulted in a significant reduction in enamel hardness for both adhesive systems. However, enamel associated with flash-free brackets retained significantly higher microhardness values than enamel bonded using conventional adhesives. This finding may be attributed to the unique polymeric structure of the flash-free adhesive system, which incorporates a low-viscosity, nano-filled resin embedded within a non-woven polypropylene fiber (20, 25, 37). This design could promote more uniform adhesive distribution, improved resin penetration, and reduced interfacial defects, thereby limiting acid diffusion and mineral loss beneath the bracket base. Gastric acid exposure resulted in the most pronounced reduction in enamel microhardness. Despite this aggressive challenge, flash-free brackets demonstrated higher microhardness values than conventional brackets. The low-viscosity resin exhibits good surface tension, allowing effective wetting of the enamel surface and formation of a uniform fillet around the bracket base, rather than irregular adhesive accumulations (38). These findings may contribute to improved enamel integrity under simulated experimental conditions, owing to increased polymer network stability and lower water sorption at the enamel–adhesive interface.

A limitation of this study is that it was conducted *in vitro*, and the impact of microbial flora on adhesive performance was not assessed. Consequently, caution should be exercised when extrapolating these results to *in vivo* clinical conditions. Although artificial cariogenic and gastric acid challenges were applied using standardized protocols to simulate clinical scenarios; however, the frequency, duration, and buffering capacity of such acid exposures may vary among patients. Also, microhardness assessment was limited to a single subsurface depth beneath the bracket base. Although this depth was selected based on previous studies, evaluating multiple depths could provide a more comprehensive understanding of enamel demineralization patterns. The cariogenic protocol used in the study represents a controlled, accelerated laboratory model and does not fully reproduce the dynamic oral environment. Furthermore, it is worth to mention that although the sample size was consistent with comparable *in vitro* orthodontic studies, the findings should be interpreted within the limitations of the experimental design. Future studies with larger sample sizes and predefined power calculations are recommended to confirm these results. Therefore, extrapolation of the findings to clinical conditions should be made with caution. Also, the gastric acid model represents a high-frequency, severe exposure model (e.g., severe GERD/bulimia) rather than average reflux patterns; therefore, the findings should not be generalized to average reflux exposure patterns. Future investigations should incorporate thermocycling alongside acidic challenges to better simulate intraoral conditions. In addition, evaluating cross-sectional microhardness at multiple subsurface depths would provide a more comprehensive assessment of lesion progression and enamel integrity.

## 5 Conclusion

Within the limitations of this study, flash-free polymeric adhesive systems demonstrated better enamel integrity compared with conventional adhesive systems following debonding. Under cariogenic and erosive challenges, flash-free brackets exhibited higher enamel cross-sectional microhardness and distinct adhesive remnant patterns. These findings suggest that flash-free polymeric adhesive systems may provide improved enamel protection during orthodontic treatment, particularly in patients exposed to cariogenic or gastric acid challenges.

## Data Availability

The original contributions presented in the study are included in the article/Supplementary Material, further inquiries can be directed to the corresponding authors.
